# Associations between Bronchopulmonary Dysplasia, Insulin-like Growth Factor I and Nutrition

**DOI:** 10.3390/nu16070957

**Published:** 2024-03-27

**Authors:** Dana F. J. Yumani, Floor H. Walschot, Harrie N. Lafeber, Mirjam M. van Weissenbruch

**Affiliations:** Division of Neonatology, Department of Pediatrics, Location VU University Medical Center, Amsterdam University Medical Centers, De Boelelaan 1117, 1081 HV Amsterdam, The Netherlands; f.walschot@amsterdamumc.nl (F.H.W.); m.vanweissenbruch@amsterdamumc.nl (M.M.v.W.)

**Keywords:** insulin-like growth factor I, bronchopulmonary dysplasia, preterm infants, nutrition, donor human milk

## Abstract

Insulin-like growth factor I (IGF-I) has been suggested as an important factor in the pathogenesis of bronchopulmonary dysplasia (BPD). In turn, nutrition has been associated with IGF-I levels and could be of importance in the pathogenesis of BPD. This study aimed to explore the association between nutrition, the IGF-I axis and the occurrence of BPD. Eighty-six preterm infants (44 male, mean gestational age: 29.0 weeks (standard deviation: 1.7 weeks)) were enrolled in an observational study. Serum IGF-I (µg/L) and insulin-like growth factor binding protein 3 (IGFBP-3; mg/L) were measured at birth and at 2, 4 and 6 weeks postnatal age. BPD was diagnosed at 36 weeks postmenstrual age. Twenty-nine infants were diagnosed with BPD. For every µg/L per week increase in IGF-I, the odds of BPD decreased (0.68, 95% CI 0.48–0.96, corrected for gestational age). The change in IGF-I in µg/L/week, gestational age in weeks and a week of predominant donor human milk feeding were associated with the occurrence of BPD in the multivariable analysis (respectively, OR 0.63 (0.43–0.92), OR 0.44 (0.26–0.76) and 7.6 (1.2–50.4)). IGFBP-3 was not associated with the occurrence of BPD in the multivariable analysis. In conclusion, a slow increase in IGF-I levels and a lower gestational age increase the odds of BPD. Donor human milk might increase the odds of BPD and should be further explored.

## 1. Introduction

Bronchopulmonary dysplasia (BPD) is one of the most frequent complications following preterm birth [1,2,3]. In BPD, the preterm lung is damaged by various insults and repairs over time, causing decreased saccular and alveolar septation and disturbed vascular development in the lungs [1]. This lung injury causes a limitation in lung function which persists into adulthood [2]. Despite insights gained in the pathogenesis of BPD in the past few years [4], therapeutic options to prevent BPD have only shown a moderate reduction in the incidence of BPD [1,2]. To find more effective therapeutic options, a deeper understanding of the pathways associated with the development of BPD could lead to new vantage points for intervention strategies.

Insulin-like growth factor I (IGF-I) has been shown to have the potential to significantly reduce the incidence of BPD. A recent trial targeting the prevention of retinopathy of prematurity (ROP) by IGF-I infusion showed a concomitant decline in BPD occurrence [5]. Previous research had actually already shown that IGF-I plays a role in the development and differentiation of various lung cells [6]. Moreover, IGF-I has anti-inflammatory and anti-oxidative effects [7,8,9], which could potentially reduce lung injury. Nevertheless, IGF-I infusion is an invasive and tedious procedure. Therefore, less invasive strategies which could increase endogenous IGF-I levels may offer a more easily implementable preventive strategy.

Nutritional intake has been associated with IGF-I levels [10]. Therefore, nutrition may potentially be associated with the incidence of BPD.

This study aimed to explore the association between nutrition, the IGF-I axis and the occurrence of BPD.

## 2. Materials and Methods

### 2.1. Study Population

Preterm infants with a gestational age of 24 to 32 weeks were enrolled in the NUTRIE study, a longitudinal observational study on nutrition in relation to the endocrine regulation of preterm growth [11]. The primary objective of this study was to investigate the association between growth-related endocrine parameters, primarily IGF-I, and growth and body composition. BPD was documented as a comorbidity. The infants were born between 2015 and 2018 and were admitted to the level III neonatal intensive care unit of Amsterdam UMC (location VU University medical center).

Infants with a substantial congenital anomaly or syndrome were excluded. The study was approved by the medical research ethics committee of the VU University Medical Center (approval number: 2014.491) and was registered at the Dutch Trial Register [11]. The study was conducted in line with good clinical practice guidelines and the Declaration of Helsinki.

Power calculations were carried out for the primary outcome of the NUTRIE study. No power calculations were carried out for the results presented in this paper.

### 2.2. Measures of Endocrine Parameters

Blood was drawn every other week up to 36 weeks postmenstrual age (PMA) for the measurement of IGF-I and IGFBP-3. Analyses of IGF-I were conducted using chemiluminescence immunoassays (LIAISON^®^, DiaSorin, Saluggia, Italy) with an intra-assay percent coefficient of variation (%CV) of 8% and an inter-assay %CV of 7%. IGFBP-3 was analyzed with a sandwich ELISA (DRG Instruments GmbH, Marburg, Germany) with an intra-assay %CV of 5% and an inter-assay %CV of 13%.

### 2.3. Morbidities

BPD was defined as the need for supplemental oxygen at 36 weeks PMA. Grade 1 BPD was defined as respiratory support through a nasal cannula with a flow of 2 L/min or less, grade 2 as a flow of 2 L/min or more through a nasal cannula or noninvasive positive airway pressure, and grade 3 as invasive mechanical ventilation [12]. In addition, data on comorbidities were collected (see Supplementary Materials Section S1.1).

### 2.4. Nutrition

Nutrition was provided according to the local protocol (see Supplementary Materials Section S1.2).

Data were obtained from hospital records to calculate the daily macronutrient intake based on the reference values for human milk (Table 1). Macronutrient intake, type of enteral nutrition and parenteral nutrition were assessed as potential confounders. Type of enteral nutrition was defined as predominantly own mother’s milk if at least 60% of the enteral intake consisted of own mother’s milk. Likewise, predominant donor human milk and predominant formula feeding were defined as at least 60% of enteral intake consisting of, respectively, donor human milk or formula.

### 2.5. Statistical Analysis

Comparisons between infants with and without BPD were analyzed, depending on the data distribution, with either the independent sample *t*-test or Mann–Whitney U test and the chi-square test or Fisher’s exact test. Logistic regression was used to correct variables for gestational age. A final backward logistic regression model was made, which included every variable which was significantly associated with BPD after correction for gestational age.

To limit the bias of repeated measures, the estimated change in IGF-I and IGFBP-3 per infant was calculated. A mixed model with a random intercept and slope, IGF-I (µg/L) as the dependent variable and postmenstrual age (weeks) as a covariate was used to estimate the mean change in IGF-I in µg/L/week per infant. The same was carried out for IGFBP-3 levels.

Analyses were conducted using IBM^®^ SPSS^®^ Statistics 28 for Windows (IBM Corp., Armonk, NY, USA). Two-sided statistical significance was assumed at *p*-values less than 0.05.

## 3. Results

Eighty-six infants were included in the analyses [15] (Table 2). Fourteen infants had grade 1 BPD, 15 infants had grade 2 BPD and there were no cases of grade 3 BPD.

BPD was associated with a lower gestational age, a lower birth weight, a longer duration of mechanical ventilation and a higher incidence of comorbidities (Table 2). After correction for gestational age, IRDS remained the only comorbidity associated with the occurrence of BPD: OR 4.2 (95% CI 1.4–13.0, *p* = 0.012).

**Figure 1:**
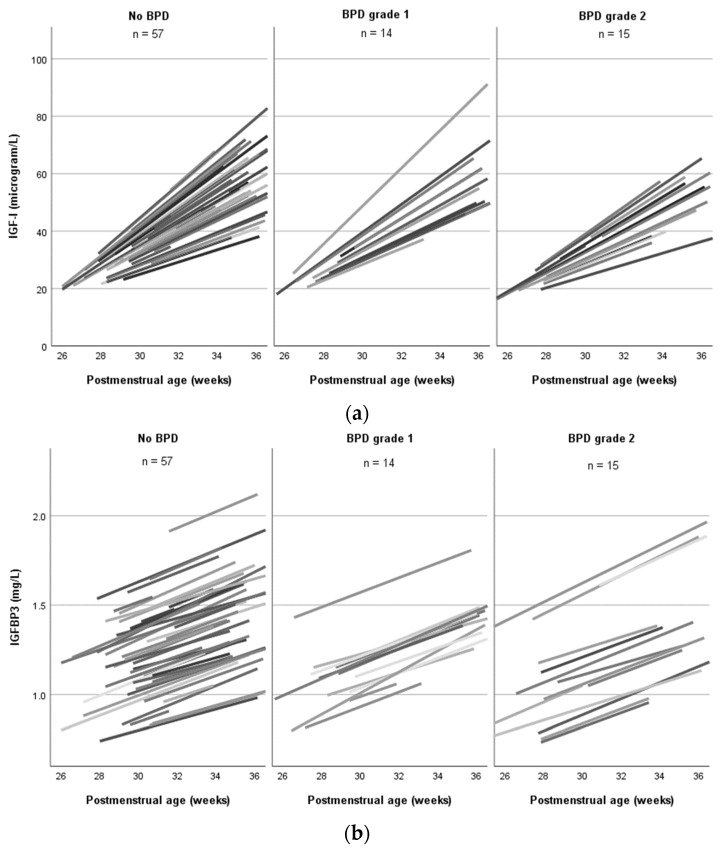
Mean change in IGF-I and IGFBP-3 serum levels in infants with and without BPD up to 36 weeks postmenstrual age. (**a**) The mean change in IGF-I was estimated using a mixed model. Each line represents one infant. (**b**) The mean change in IGFBP-3 was estimated using a mixed model. Each line represents one infant. BPD: bronchopulmonary dysplasia; IGFBP-3: insulin-like growth factor binding protein 3; IGF-I: insulin-like growth factor I.

The change in IGF-I between birth and 36 weeks PMA was not different between the infants with and without BPD (Figure 1). However, from birth through to 34 weeks PMA, there was a mean change in IGF-I of 2.8 ± 2.2 µg/L per week in infants with BPD compared to 4.5 ± 1.8 µg/L per week in infants without BPD (OR 0.68 (95% CI 0.48–0.96, *p* = 0.026; corrected for gestational age)).

The change in IGFBP-3 was not different between the infants with and without BPD (Figure 1).

**Figure 2:**
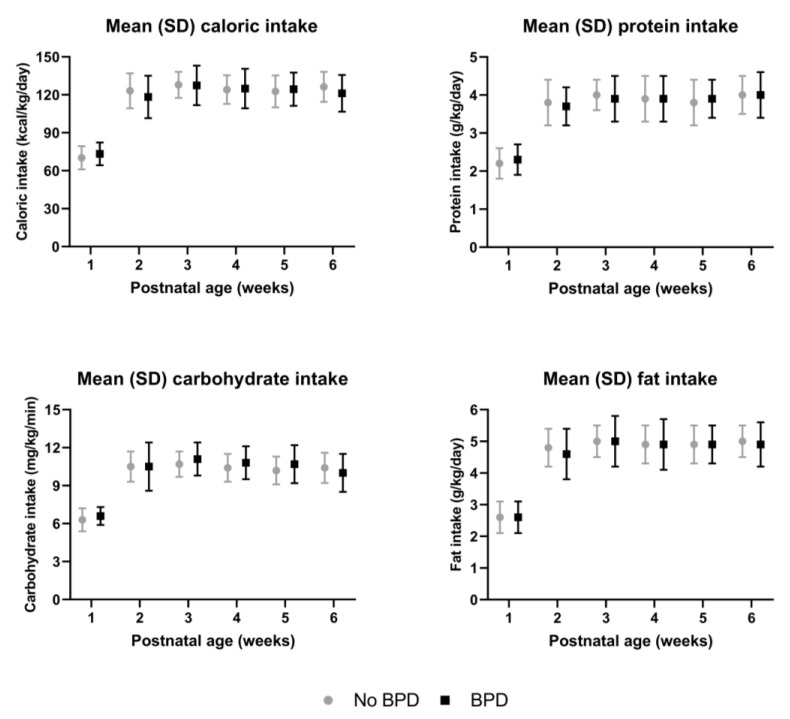
Nutritional intake in preterm infants with and without BPD up to 6 weeks postnatal age.

**Figure 3:**
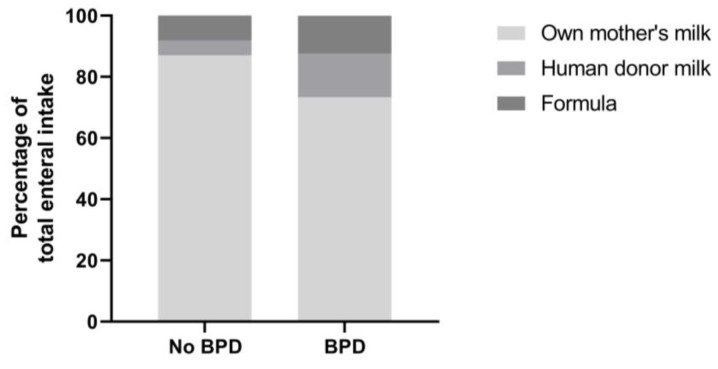
Enteral intake in preterm infants with and without BPD during hospitalization.

The infants with and without BPD had a comparable macronutrient and caloric intake during hospitalization (Figure 2). Both groups were mainly fed own their mother’s milk (Figure 3). The median duration of predominant donor human milk feeding was 4 days (IQR 0–11.5) in infants without BPD, compared to 7 days (2.5–18.5) in infants with BPD (not significant after correction for gestational age). Throughout hospitalization, 13 infants were predominantly fed donor human milk for at least a week. Of these infants, 10 (76.9%) developed BPD, compared to 19 out of 73 (26%) infants who were predominantly fed donor milk for a shorter period (*p* = 0.001). Corrected for gestational age, being predominantly fed donor milk for at least one week was associated with higher odds of BPD compared to infants fed donor milk for less than a week: OR 4.6 (95% CI 1.03–20.55, *p* = 0.046). The change in IGF-I during hospitalization was not significantly different in infants predominantly fed donor human milk for less than a week compared to those predominantly fed donor human milk for more than a week (Supplementary Materials Section S2.3). In total, two infants were predominantly fed formula. No subgroup analyses were carried out for formula feeding due to the low incidence.

After correction for gestational age, there were no significant differences in intake in infants with or without BPD.

After correction for gestational age, there were no significant differences in the type of enteral nutrition in infants with or without BPD.

The median duration of parenteral nutrition was 12 days (IQR 9–22) in infants with BPD, compared to 10 days (IQR 8–13) in infants without BPD (not significant after correction for gestational age). In the first two weeks of life, the infants with BPD had a larger proportion of their intake as parenteral nutrition compared to the infants without BPD: 37.9% (95% CI 29.4–49.5) versus 32.3% (95% CI 23.2–42.7) (not significant after correction for gestational age).

The change in IGF-I in μgram/L per week, gestational age in weeks, IRDS (yes or no) and predominantly donor human milk use for at least one week compared to less than a week were included in our final predictive model. A slow increase in IGF-I, lower gestational age and at least one week of predominantly donor human milk feeding compared to less than a week of predominantly donor human milk feeding were associated with higher odds of BPD (Table 3). Macronutrient and caloric intakes were not significant confounding factors in this association (see Supplementary Materials Section S2.4).

## 4. Discussion

This study showed that, in particular, before 35 weeks PMA, low IGF-I levels and a slower increase in IGF-I were associated with higher odds of BPD in preterm infants. IGFBP-3 showed a similar pattern but was not significant in the multivariable analysis. Gestational age and donor human milk consumption were significant confounders in the association between IGF-I and BPD.

Other studies have also shown that preterm infants with BPD have lower postnatal IGF-I levels and a slower increase in IGF-I compared to infants without BPD [16,17,18]. Also, in cases of intrauterine growth restriction, IGF-I levels are decreased and the odds of BPD increase [19,20]. Previous research has suggested that IGF-I levels are a representation of the grade of infant immaturity, and the link between IGF-I and BPD may be an indirect connection [17]. However, after adjustment for gestational age and other potential confounders, which significantly differed between the infants with and without BPD, the association remained significant. This makes a functional direct mechanism more plausible.

### 4.1. Potential Pathways through Which IGF-I and IGFBP-3 Could Influence the Occurrence of BPD

A number of factors may play a role in the association between IGF-I and BPD. IGF-I signaling influences the development and differentiation of several types of lung cells [6]. Lower IGF-I could therefore arrest lung development. Furthermore, IGF-I is known to be an important factor in lung injury and repair processes. During lung injury, IGF-I increases the proliferation of lung fibroblasts and enhances collagen products [16,21]. In addition, pre- and postnatal inflammation contributes to lung injury and, subsequently, to the development of BPD. Previous studies have shown that the incidence of BPD increases in the case of sepsis due to increased oxidative stress, inflammation and endothelial lung injury [3,22]. IGF-I is known to have a protective effect against inflammation and to show anti-oxidative effects, protecting cells from oxidative-stress-induced apoptosis [7,8,9]. The protective effect against inflammation and the anti-oxidative effects of IGF-I may be important in decreasing the risk of BPD. In support of this, a recent study aiming to reduce the occurrence of ROP by administering human recombinant IGF-I showed that an increase in pro-inflammatory cytokines, e.g., interleukin-6, was associated with a subsequent decrease in IGF-I, and IGF-I administration reduced the occurrence of BPD [5,23].

IGFBP-3 is the main binding protein for IGF-I and determines the bioavailability of IGF-I. Thus, it could be hypothesized that IGFBP-3 indirectly relates to BPD through IGF-I. Hypothesizing further, if the association between IGFBP-3 and BPD is mainly indirect, it would be statistically weaker than the direct association between IGF-I and BPD. However, it has been stated that IGFBP-3 on its own also downregulates inflammation and could thus directly be associated with BPD [24]. Nevertheless, in line with our results, previous studies did not find an association between IGFBP3 levels and BPD in preterm infants, while they did find an association with IGF-I [16]. Speculatively, this may be due to lower statistical power because the range of IGFBP-3 levels was relatively small.

### 4.2. Possible Interactions between Nutrition, IGF-I Levels and BPD

In line with others, in our study, the infants with BPD had a comparable macronutrient intake to the infants without BPD [16]. Milanesi and colleagues reported that infants who developed BPD received less than the recommended daily protein/energy ratio [25]. Nevertheless, in our population, the infants reached the recommended daily intake by the second week of life. However, our study did show that the infants who were predominantly fed donor human milk for at least a week had higher odds of BPD compared to those who were predominantly fed donor human milk for a shorter period. This could be due to the lower energy and macronutrient content of donor human milk compared to own mother’s milk. Nevertheless, in our study population, nutrient intake was not a confounding factor in the association between donor human milk intake and BPD. On the other hand, it has been described that human milk contains IGF-I, and human milk IGF-I levels have been associated with growth in term infants [26]. Furthermore, Holder pasteurization can reduce IGF-I levels by up to 40% [27]. Therefore, it could be hypothesized that donor human milk could lead to lower IGF-I levels in preterm infants. In addition to a possible direct uptake of IGF-I from human milk, it has been described that micronutrients and branched amino acids in human milk may stimulate the infant’s IGF-I axis [28]. Pasteurization has been reported to affect these factors [29], thus supporting a potential negative effect of pasteurized donor human milk on endogenous IGF-I levels. In the future, novel techniques such as high-temperature–short-time pasteurization, high-pressure processing and ultraviolet C irradiation may offer alternatives to Holder pasteurization [30]. It is noteworthy, however, that in our population, the rise in IGF-I was not significantly different in the infants predominantly fed donor human milk for less than a week compared to those predominantly fed donor human milk for more than a week. This suggests that a possible relationship between donor human milk and BPD may not be through a direct effect on IGF-I.

However, in our study, the participants were mainly fed their own mother’s milk. The absolute quantity of donor human milk was small and did not correlate with the occurrence of BPD. Importantly, the infants predominantly fed donor human milk for at least a week had a lower gestational age and birth weight compared to the infants predominantly fed donor human milk for less than a week (see Supplementary Materials Section S2.2). Therefore, our findings remain speculative.

### 4.3. Strengths and Limitations

Data on actual nutrient intake, as opposed to the prescribed amount, were collected prospectively, and changes in IGF-I over time were calculated for every participant. Nevertheless, blood samples were taken every other week, leading to a relatively low sample size in the analyses per week PMA.

Our population showed a relatively high BPD incidence. The definition used for BPD categorizes BPD severity according to respiratory support at 36 weeks PMA. This could lead to the inclusion of infants who have had a relatively short duration of respiratory support [12]. Previous studies and several clinics still use the older definition criteria, which define BPD as having had a need for supplemental oxygen for at least 28 days [31]. Using the older criteria, eight infants would no longer have been classified as having BPD, while nine infants classified as not having BPD would have been classified as otherwise. Using the definition by Jobe et al., our results still showed a similar pattern with regards to the relationship between IGF-I, BPD and nutrition. In addition, the incidence in our population would have stayed relatively high despite average antenatal corticosteroid use [32] and a short duration of mechanical ventilation.

Moreover, only a limited number of extremely preterm infants were enrolled, limiting the statistical power of the analyses below 30 weeks PMA. In addition, multiple tests in post hoc analyses increased the risk of type I errors. Lastly, it remains noteworthy that causality could not be examined due to the observational nature of this study.

## 5. Conclusions

This study provided further insight into the relationship between the development of the endocrine axis and the occurrence of BPD: low IGF-I levels and a slower increase in IGF-I are associated with the occurrence of BPD. Furthermore, this study pointed towards a possible effect of donor human milk on the development of BPD. However, more research is needed to investigate whether there truly is an increased risk of BPD in infants fed donor human milk.

## Figures and Tables

**Table 1:** Reference values used for the nutritional composition of human milk per 100 mL.

	OMM	OMM + BMF (4.4 g/100 mL)	DHM	DHM + BMF (4.4 g/100 mL)
Energy (kcal)	68.5	83.8	60	75
Protein (g)	1.5	2.6	0.8	1.9
Carbohydrates (g)	7.3	10.0	7.5	10.2
Fat (g)	3.3	3.3	2.9	2.9

BMF: breast milk fortifier; DHM: donor human milk; OMM: own mother’s milk. Reference values for OMM were derived from meta-analyses [13,14]. Donor human milk composition was based on analyses of the donor milk batches administered to the first 23 study participants.

**Table 2:** Baseline characteristics.

	All (*n* = 86) ^a^	BPD (*n* = 29)	No BPD (*n* = 57)	*p* Value
Gender, *n* male (%)	44 (51.2)	17 (58.6)	27 (47.4)	0.324 ^b^
Ethnicity, *n* white (%)	65 (75.6)	22 (75.9)	43 (75.4)	0.966 ^b^
Gestational age (weeks), mean (SD)	29.0 (1.7)	27.9 (1.7)	29.6 (1.5)	**<0.001 ^c^**
Birthweight (g), mean (SD)	1217 (312)	1055 (271)	1298 (301)	**0.001 ^c^**
Birthweight SDS, mean (SD)	0.0 (0.7)	0.0 (0.7)	0.0 (0.7)	0.765 ^c^
Birthweight SDS < −1.3, *n* (%)	3 (3.5)	1 (3.4)	2 (3.5)	1.000 ^d^
Antenatal steroids ^e^, *n* (%)	56 (65.1)	17 (58.6)	39 (68.4)	0.473 ^b^
Postnatal steroids ^f^, *n* (%)	8 (9.3)	5 (17.2)	3 (5.3)	0.113 ^d^
Ventilation days, median (IQR)	0 (0.0–5.0)	2.0 (0.0–9.0)	0.0 (0.0–2.3)	**0.007 ^g^**
IRDS, *n* (%)				**<0.001 ^b^**
IRDS stage I–II	24 (27.9)	14 (48.3)	10 (17.5)	
IRDS stage III–IV	19 (22.1)	9 (31.0)	10 (17.5)	
ROP, *n* (%)				0.532 **^d^**
ROP stage I	4 (4.7)	1 (3.4)	3 (5.3)	
ROP stage III	1 (1.2)	1 (3.4)	0 (0.0)	
PDA requiring treatment, *n* (%)	8 (9.3)	5 (17.2)	3 (5.3)	0.113 ^d^
NEC, *n* (%)	6 (7.0)	4 (13.8)	2 (3.5)	0.173 ^d^
LOS, *n* (%)	30 (34.9)	14 (48.3)	16 (28.1)	0.063 ^b^
IVH grade ≥ III, *n* (%)	3 (3.5)	3 (10.3)	0 (0)	**0.036 ^d^**
PHVD, *n* (%)	8 (9.3)	3 (10.3)	5 (8.8)	1.000 **^d^**
PVL, *n* (%)	3 (3.5)	1 (3.4)	2 (3.5)	1.000 **^d^**

^a^ see Supplementary Materials for inclusion flow chart (Section S2.1). ^b^ chi-square test. ^c^ independent sample *t*-test. ^d^ Fisher’s exact test. ^e^ Antenatal steroids were defined as at least 2 doses of bethametason. ^f^ Postnatal steroids were defined as at least 3 days of hydrocortisone treatment. ^g^ Mann–Whitney U test. Values in bold are statistically significant. BPD: bronchopulmonary dysplasia, IRDS: infant respiratory stress syndrome, IVH: intraventricular hemorrhage, LOS: Late-onset sepsis; NEC: necrotizing enterocolitis; PDA: patent ductus arteriosus, PHVD: posthemorrhagic ventricular dilatation, PVL: periventricular leukomalacia, ROP: retinopathy of prematurity, SD: standard deviation, SDS: standard deviation score.

**Table 3:** Multivariable logistic regression for the occurrence of BPD.

	B (SE)	*p*-Value	Odds Ratio (95% CI)
Included variables			
Constant	23.9 (8.0)	0.003	
Change in IGF-I (µg/L per week)	−0.5 (0.2)	0.018	0.63 (0.43–0.92)
Gestational age at birth (weeks)	−0.8 (0.3)	0.003	0.44 (0.26–0.76)
Predominantly donor human milk for at least 1 week ^a^	2.0 (1.0)	0.035	7.6 (1.2–50.4)

R^2^ = 0.358 (Cox and Snell) and 0.498 (Nagelkerke). Model χ^2^ (3) = 26.63, *p* < 0.001. ^a^ Predominantly donor human milk for at least one week, compared to less than one week of predominantly donor human milk feeding. Predominant donor milk feeding was defined as at least 60% of total enteral intake consisting of donor human milk. IRDS was the only variable removed in backward regression. IGF-I: insulin-like growth factor I; IRDS: infant respiratory distress syndrome.

## Data Availability

The data presented in this study are available upon request from the corresponding author.

## Supplementary Materials

The following supporting information can be downloaded at https://www.mdpi.com/article/10.3390/nu16070957/s1: The Supplementary Materials contain the following data: Section S1.1: Definitions of diseases included as clinical data. Section S1.2: Local nutrition protocol. Section S2.1: Inclusion/drop-out flow chart. Section S2.2: Baseline characteristics of the predominantly donor human milk-fed group. Section S2.3: Association between donor human milk intake and IGF-I levels. Section S2.4: Association between donor human milk intake and BPD corrected for nutrition.
