# Supplementary material for: Associations between Bronchopulmonary Dysplasia, Insulin-like Growth Factor I and Nutrition
Source: Nutrients. 2024 Mar 27;16(7):957. doi: 10.3390/nu16070957 (PMC11013493; doi:10.3390/nu16070957)
Supplement: Supplementary file 1 [file nutrients-16-00957-s001.zip › nutrients-2890565-supplementary.pdf]

S1. Methods

S1.1. Definitions of Diseases Included as Clinical Data

Necrotizing enterocolitis was defined according to the modified Bell criteria (Walsh 1986).

I. **Stage 1A Suspected NEC** based on temperature instability, apnea, bradycardia, or lethargy combined with gastric retention, abdominal distention, emesis, or heme-positive stool and normal or mild intestinal dilation, or mild ileus on radiography.
II. **Stage 1B Suspected NEC** based on temperature instability, apnea, bradycardy, or lethargy combined with grossly bloody stool and normal or mild intestinal dilation, or mild ileus on radiography.
III. **Stage 2A definite NEC (mildly ill)** based on temperature instability, apnea, bradycardy, or lethargy combined with grossly bloody stool and absent bowel sounds with or without abdominal tenderness and Intestinal dilation, ileus, or pneumatosis intestinalis on radiography.
IV. **Stage 2B definite NEC (moderately ill)** based on temperature instability, apnea, bradycardy, or lethargy, as well as mild metabolic acidosis and thrombocytopenia, combined with grossly bloody stool and absent bowel sounds, definite tenderness, with or without abdominal cellulitis or right lower quadrant mass and Intestinal dilation, ileus, pneumatosis intestinalis and ascites on radiography.
V. **Stage 3A advanced NEC (severely ill, intact bowel)** based on temperature instability, severe apnea, bradycardia, hypotension, lethargy, respiratory and metabolic acidosis, disseminated intravascular coagulation, and neutropenia combined with grossly bloody stool and absent bowel sounds, signs of peritonitis, marked tenderness, and abdominal distention, with or without abdominal cellulitis or right lower quadrant mass and Intestinal dilation, ileus, pneumatosis intestinalis and ascites on radiography.
VI. **Stage 3B advanced NEC (severely ill, perforated bowel)** based on temperature instability, severe apnea, bradycardia, hypotension, lethargy, respiratory and metabolic acidosis, disseminated intravascular coagulation, and neutropenia combined with grossly bloody stool and absent bowel sounds, signs of peritonitis, marked tenderness, and abdominal distention, with or without abdominal cellulitis or right lower quadrant mass and Intestinal dilation, ileus, pneumatosis intestinalis, ascites and pneumoperitoneum on radiography.

Walsh MC, Kliegman RM. Necrotizing enterocolitis: treatment based on staging criteria. Pediatr Clin North Am 1986;33(1):179-201. doi: 10.1016/s0031-3955(16)34975-6 [published Online First: 1986/02/01]

Late onset sepsis was defined as sepsis occurring 72 hours after birth with a positive blood culture or clinical signs of sepsis and relevant antimicrobial treatment (Dong 2015).

Dong Y, Speer CP. Late-onset neonatal sepsis: recent developments. Arch Dis Child Fetal Neonatal Ed 2015;100(3):F257-F63. doi: 10.1136/archdischild-2014-306213 [published Online First: 2014/11/25]

Intraventricular hemorrhage and **post hemorrhagic ventricle dilatation** were defined according to Papile (Papile 1978).

I. Grade 1: subependymal hemorrhage
II. Grade 2: intraventricular hemorrhage without ventricular dilatation
III. Grade 3: intraventricular hemorrhage with ventricular dilation
IV. Grade 4: intraventricular hemorrhage with parenchymal hemorrhage

Papile LA, Burstein J, Burstein R, et al. Incidence and evolution of subependymal and intraventricular hemorrhage: a study of infants with birth weights less than 1,500 gm. J Pediatr 1978;92(4):529-34. doi: 10.1016/s0022-3476(78)80282-0 [published Online First: 1978/04/01]

Retinopathy of prematurity was defined according to the 2005 International Classification of Retinopathy of Prematurity

I. Stage I: Demarcation line
II. Stage 2: Ridge
III. Stage 3: Extraretinal fibrovascular proliferation, i.e. neovascularization from the ridge into the vitreous
IV. Stage 4: Partial retinal detachment

International Committee for the Classification of Retinopathy of P. The International Classification of Retinopathy of Prematurity revisited. Arch Ophthalmol 2005;123(7):991-9. doi: 10.1001/archopht.123.7.991 [published Online First: 2005/07/13]

Infant respiratory distress syndrome was graded according to Giedion (Giedion 1973).

I. Grade 1: Reticulogranular pattern
II. Grade 2: Reticulogranular patterin and air bronchogram
III. Grade 3: Reticulogranular pattern, air bronchogram and loss of distinct heart borders
IV. Grade 4: Diffuse opacification and obscuration of the cardiac silhouette

Giedion A, Haefliger H, Dangel P. Acute pulmonary X-ray changes in hyaline membrane disease treated with artificial ventilation and positive end-expiratory pressure (PEP). Pediatr Radiol 1973;1(3):145-52. doi: 10.1007/BF00974058 [published Online First: 1973/10/01]

Patent ductus arteriosus was defined as a patent ductus on ultrasound which was treated with medication or required surgical intervention. Treatment was considered when an infant had respiratory distress, a patent ductus with left-right shunting and:

- 3 characteristics of a moderate left-right shunting with a duct diameter of at least 1.6 mm and a pulsatile flow pattern
OR

- 2 characteristics of a large left-right shunt and a duct diameter of at least 2 mm and a pulsatile flow pattern

Table S1. Patent ductus arteriosus classification.

	**Small Left-Right Shunt**	**Moderate Left-Right Shunt**	**Large Left-Right Shunt**
**Diameter of the ductus arteriosus (mm)**	<1.6	1.6–2.0	>2.0
**Flow pattern in the ductus arteriosus**	Closing	Growing/pulsatile	Pulsatile
**End diastolic flow in left pulmonary artery (m/s)**	<0.20	0.20–0.40	>0.40
**Flow in the aorta descendens**	Antegrade flow	No or minimally reversed flow	Reversed flow
**Flow speed in ductus arteriosus**	>2.0	1.0–2.0	<1.0
**Left atrial to aortic root ration**	<1.4	1.4–1.6	>1.6

Source: local protocol Amsterdam University Medical Centers Neonatal Intensive Care Unit

S1.2. Local Nutrition Protocol

Nutrition was initially provided through total parenteral nutrition and minimal enteral feeding.[1] Within seven to ten days, 160 ml/kg/day human milk was scheduled to be given fortified with breast milk fortifier. Either own mother's milk or donor human milk was given. Donor human milk underwent Holder pasteurization (heated at 62.0°C to 62.5°C for 30 minutes, followed by fast cooling to under 4°C).[2] In the case of poor growth, up to 2% Nenatal Human Milk Protein Fortifier (Nutricia, Wageningen, The Netherlands) or fat emulsion Calogen (Nutricia, Wageningen, The Netherlands), was added to the fortified human milk. If own mother's milk was not available, donor human milk was supplied up to 32 weeks PMA, followed by preterm starters formula till discharge home. If parents declined the use of donor human milk, infants were fed preterm starters formula from birth.

[1] Yumani DFJ, Calor AK, van Weissenbruch MM. The Course Of IGF-1 Levels and Nutrient Intake in Extremely and Very Preterm Infants During Hospitalisation. *Nutrients* 2020;12(3) doi: 10.3390/nu12030675 [published Online First: 2020/03/07]

[2] de Waard M, Mank E, van Dijk K, et al. Holder-Pasteurized Human Donor Milk: How Long Can It Be Preserved? *J Pediatr Gastroenterol Nutr* 2018;66(3):479-83. doi: 10.1097/MPG.0000000000001782 [published Online First: 2017/10/12]

S2. Results

S2.1. Inclusion/Drop-Out Flow Chart

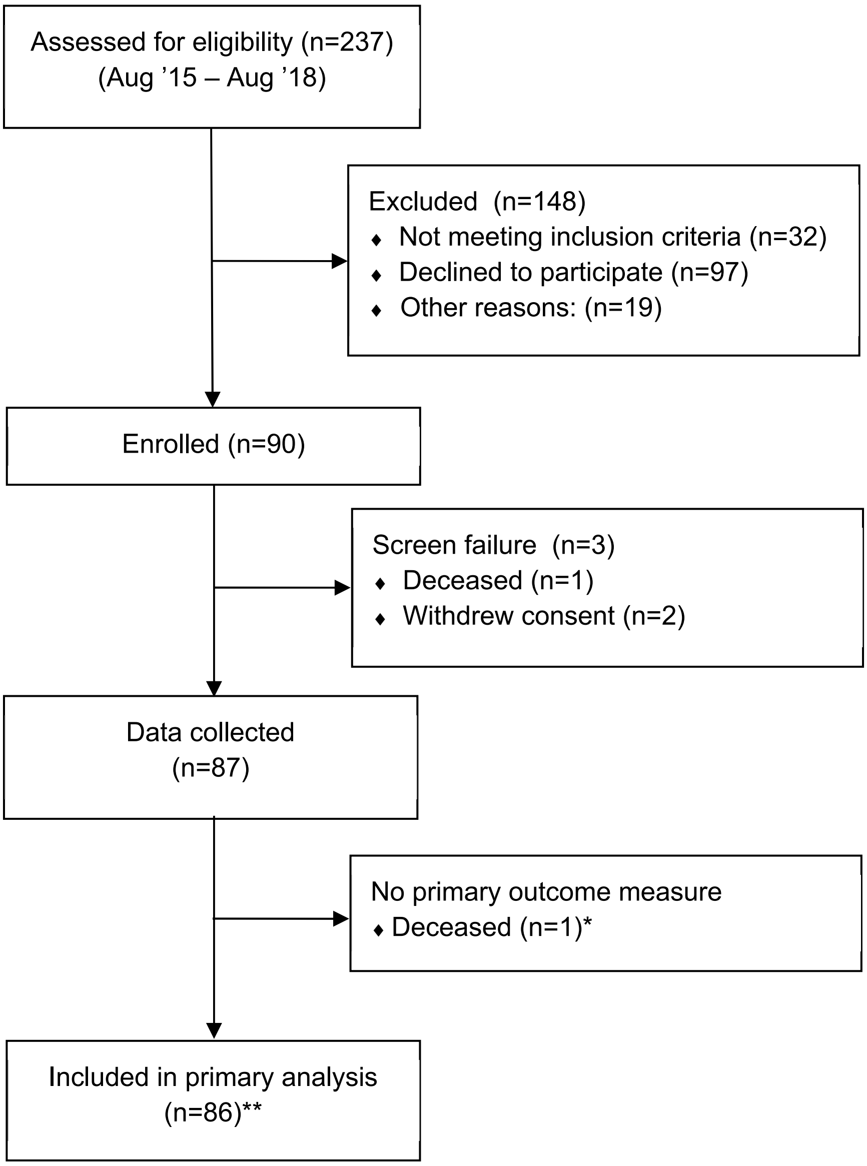

Figure S1. Inclusion flow chart.

* The infant was born at 24 weeks gestational age and deceased in the third week of life. Reason of death was a progressive PHVD, combined with a NEC stage IIA and severe pulmonary interstitial emphysema.

** All 86 participants were included in the analyses presented in the study. 6 infants discontinued study participation before term age (study burden too high (n=5), unresponsive to term age follow-up invite), but permission was given to continue data collection from the file and blood draws were done before study discontinuation.

S2.2. Baseline Characteristics in Light of Donor Human Milk Consumption

Table S2. Baseline characteristics in infants predominantly fed donor human milk for 7 days or more versus less than 7 days.

	All (n = 86)	DHM Fed [a] ≥ 7 Days (n = 13)	DHM Fed < 7 Days (n = 73)	*p* Value
**Gender, n male (%)**	44 (51.2)	7 (53.8)	27 (50.7)	1.000[b]
**Ethnicity, n white (%)**	65 (75.6)	10 (76.9)	55 (75.3)	1.000[b]
**Gestational age (weeks), mean (SD)**	29.0 (1.7)	27.4 (1.6)	29.3 (1.6)	**<0.001**[c]
**Birthweight (g), mean (SD)**	1217 (312)	960 (183)	1262 (309)	**0.001**[c]
**Birthweight SDS, mean (SD)**	0.0 (0.7)	-0.1 (0.8)	0.0 (0.7)	0.627[c]
**Birthweight SDS < -1.3, n (%)**	3 (3.5)	0 (0.0)	3 (4.1)	1.000[d]
**Antenatal steroids [e], n (%)**	56 (65.1)	5 (38.5)	51 (69.9)	0.054[d]
**Postnatal steroids [f], n (%)**	8 (9.3)	3 (23.1)	5 (6.8)	0.097[d]
**Ventilation days, median (IQR)**	0 (0.0 - 5.0)	1.0 (0.0 – 14.0)	0.0 (0.0 – 4.5)	0.263[g]
**IRDS, n (%)**				0.117[d]
**IRDS stage I–II**	24 (27.9)	7 (53.8)	17 (23.3)	
**IRDS stage III–IV**	19 (22.1)	2 (15.4)	17 (23.3)	
**ROP, n (%)**				0.569[d]
**ROP stage I**	4 (4.7)	1 (7.7)	3 (4.1)	
**ROP stage III**	1 (1.2)	0 (0.0)	1 (1.4)	
**PDA requiring treatment, n (%)**	8 (9.3)	2 (15.4)	6 (8.2)	0.347[d]
**NEC, n (%)**	6 (7.0)	3 (23.1)	3 (4.1)	**0.042**[d]
**LOS, n (%)**	30 (34.9)	6 (46.2)	24 (32.9)	0.363[b]
**IVH grade ≥ III, n (%)**	3 (3.5)	2 (15.4)	1 (1.4)	0.058[d]
**PHVD, n (%)**	8 (9.3)	2 (15.4)	6 (8.2)	0.347[d]
**PVL, n (%)**	3 (3.5)	1 (7.7)	2 (2.7)	0.392z[d]

[a] Donor human milk defined as at least 60% of enteral intake

[b] Chi-square Test

[c] Independent sample T-test

[d] Fisher-Exact test

[e] Antenatal steroids were defined as at least 2 doses of betamethasone

[f] Postnatal steroids were defined as at least 3 days of hydrocortisone treatment

[g] Mann-whitney U test

Values in bold are statistically significant

BPD: Bronchopulmonary dysplasia, DHM: donor human milk, IRDS: Infant respiratory stress syndrome, IVH: intraventricular hemorrhage, LOS: Late-onset sepsis; NEC: Necrotizing enterocolitis; PDA: patent ductus arteriosus, PHVD: post-hemorrhagic ventricular dilatation, PVL: periventricular leukomalacia, ROP: retinopathy of prematurity, SD: standard deviation, SDS: standard deviation score

S2.3. Association between donor human milk intake and IGF-I levels

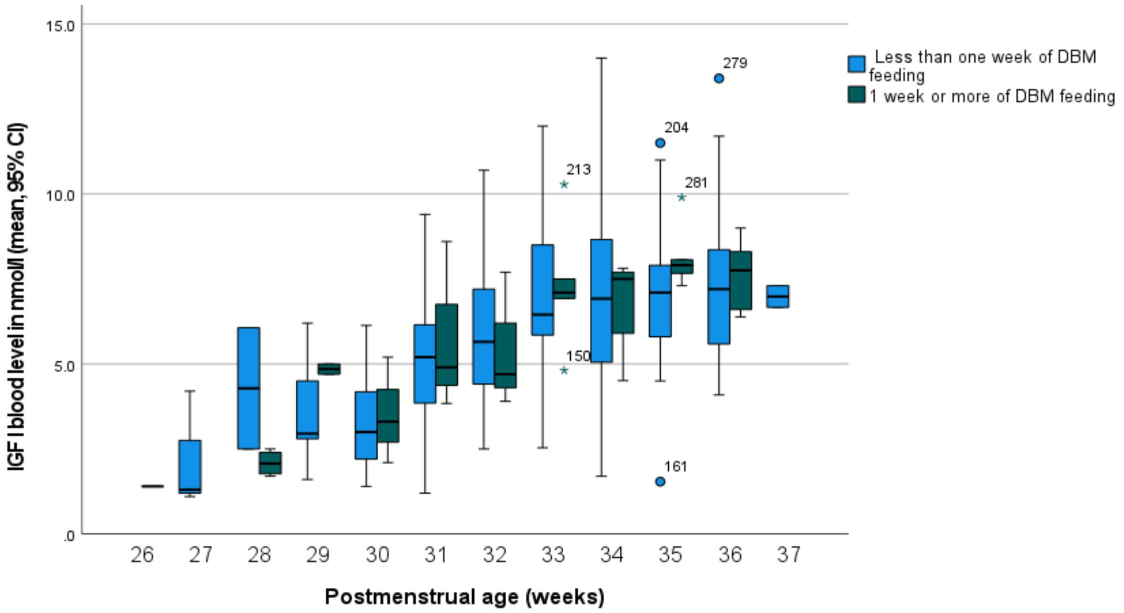

Figure S2. IGF-I levels in preterm infants predominantly fed donor human milk for 7 days or more versus less than 7 days. Eighty-six infants are included in this graph. The numerals represent outliers; each number represents an individual study participant.

The postnatal change in IGF-I did not differ between infants predominantly fed donor human milk for less than one week compared to those predominantly fed donor human milk for one week or more. (mean 3.9 (1.0) vs 4.0 (0.7) respectively, p 0.782)

S2.4. Association between Donor Human Milk Intake and BPD Corrected for the Nutrient Intake

Table S3. Association between donor human milk, protein intake and BPD.

	B (SE)	*p*-Value	Odds Ratio (95% CI)
**Included Variables**			
**Constant**	-6.2 (2.7)	0.021	
**Predominant donor human milk for at least 1 week**[a]	2.7 (0.8)	0.001	15.4 (3.3 – 72.2)
**Protein intake (g/kg/day)**	1.4 (0.7)	0.051	4.0 (1.0 – 16.4)

R^2 = 0.174 (Cox & Snell), 0.241 (Nagelkerke). Model χ^2 (2) = 16.458, p < 0.001

[a] Predominant donor human milk for at least one week compared to less than one week predominant donor human milk feeding. Predominant donor milk feeding was defined as at least 60% of total enteral intake consisting of donor human milk.

Table S4. Association between donor human milk, carbohydrate intake and BPD.

	B (SE)	*p*-Value	Odds Ratio (95% CI)
**Included variables**			
**Constant**	-4.1 (3.6)	0.255	
**Predominant donor human milk for at least 1 week**[a]	2.7 (0.7)	0.002	8.8 (2.2 – 35.9)
**Carbohydrate intake (mg/kg/min)**	0.3 (0.4)	0.394	1.4 (0.7 – 2.8)

R^2 = 0.140 (Cox & Snell), 0.194 (Nagelkerke). Model χ^2 (2) = 12.945, p 0.002

[a] Predominant donor human milk for at least one week compared to less than one week predominant donor human milk feeding. Predominant donor milk feeding was defined as at least 60% of total enteral intake consisting of donor human milk.

Table S5. Association between donor human milk, fat intake and BPD.

	B (SE)	*p*-Value	Odds Ratio (95% CI)
**Included variables**			
**Constant**	-2.8 (2.6)	0.283	
**Predominant donor human milk for at least 1 week**[a]	2.3 (0.7)	0.002	9.5 (2.3 – 38.9)
**Fat intake (g/kg/day)**	0.4 (0.6)	0.496	1.5 (0.5 – 4.5)

R^2 = 0.137 (Cox & Snell), 0.190 (Nagelkerke). Model χ^2 (2) = 12.659, p 0.002

[a] Predominant donor human milk for at least one week compared to less than one week predominant donor human milk feeding. Predominant donor milk feeding was defined as at least 60% of total enteral intake consisting of donor human milk.

Table S6. Association between donor human milk, caloric intake and BPD.

	B (SE)	*p*-Value	Odds Ratio (95% CI)
**Included variables**			
**Constant**	-5.9 (3.8)	0.003	
**Predominant donor human milk for at least 1 week**[a]	2.5 (0.8)	0.001	12.2 (2.7 – 54.4)
**Caloric intake (kcal/kg/day)**	0.0 (0.0)	0.198	1.0 (1.0 – 1.1)

R^2 = 0.137 (Cox & Snell), 0.190 (Nagelkerke). Model χ^2 (2) = 12.659, p 0.002

[a] Predominant donor human milk for at least one week compared to less than one week predominant donor human milk feeding. Predominant donor milk feeding was defined as at least 60% of total enteral intake consisting of donor human milk.

Table S7. Multivariable logistic regression for the occurrence of BPD including nutrient intake.

	B (SE)	*p*-Value	Odds Ratio (95% CI)
**Included variables**			
**Constant**	37.9 (13.9)	0.006	
**Change in IGF-I (µgram/L per week)**	-0.4 (0.2)	0.040	0.67 (0.45 – 0.98)
**Gestational age at birth (weeks)**	-1.0 (0.4)	0.006	0.38 (0.19 – 0.76)
**Predominant donor human milk for at least 1 week**[a]	2.7 (1.1)	0.014	15.3 (1.72 – 136.53)
**IRDS**	1.5 (0.9)	0.111	4.47 (0.71-28.16)
**Mean fat intake (g/kg/day)**	-2.3 (1.2)	0.066	0.10 (0.01 – 1.16)

R^2 = 0.428 (Cox & Snell), 0.595 (Nagelkerke). Model χ^2 (5) = 33.53, $p < 0.001$

[a] Predominant donor human milk for at least one week compared to less than one week predominant donor human milk feeding. Predominant donor milk feeding was defined as at least 60% of total enteral intake consisting of donor human milk.

When caloric, protein and carbohydrate intake were included in the model, the terms for the nutrient intake and IRDS were removed in the backward regression, resulting in the same regression model as depicted in table 3 of the manuscript.
